# HER2 amplification level by in situ hybridization predicts survival outcome in advanced HER2-positive breast cancer treated with pertuzumab, trastuzumab, and docetaxel regardless of HER2 IHC results

**DOI:** 10.1186/s13058-023-01746-w

**Published:** 2023-12-14

**Authors:** Jeongmin Seo, Jiwon Koh, Dae-Won Lee, Jinyong Kim, Han Suk Ryu, Kyung-Hun Lee, Tae-Yong Kim, Seock-Ah Im

**Affiliations:** 1 Department of Internal Medicine, Seoul National University Hospital, 101 Daehak-ro, Jongno-gu, Seoul, 03080 Republic of Korea; 2 Department of Internal Medicine, Seoul National University Bundang Hospital, Seongnam-si, Gyeonggi-do Republic of Korea; 3 Department of Pathology, Seoul National University Hospital, Seoul, Republic of Korea; 4 Translational Medicine, Seoul National University College of Medicine, Seoul, Republic of Korea; 5 Department of Internal Medicine, Seoul National University College of Medicine, Seoul, Republic of Korea; 6 Cancer Research Institute, Seoul National University, Seoul, Republic of Korea

**Keywords:** HER2 positive breast cancer, HER2/CEP17 ratio, HER2 copy number, HER2 in situ hybridization, Dual HER2 blockade

## Abstract

**Background:**

The role of HER2 amplification level in predicting the effectiveness of HER2-directed therapies has been established. However, its association with survival outcomes in advanced HER2-positive breast cancer treated with dual HER2-blockade remains unexplored.

**Methods:**

This is a single-center retrospective study of patients with advanced HER2-positive breast cancer treated with first-line pertuzumab, trastuzumab, and docetaxel. The primary objective was to ascertain the relationship between treatment outcomes and the level of HER2 amplification by in situ hybridization (ISH).

**Results:**

A total of 152 patients were included with a median follow-up duration of 50.0 months. Among the 78 patients who received ISH, a higher HER2/CEP17 ratio correlated significantly with longer PFS (HR 0.50, *p* = 0.022) and OS (HR 0.28, *p* = 0.014) when dichotomized by the median. A higher HER2 copy number also correlated significantly with better PFS (HR 0.35, *p* < 0.001) and OS (HR 0.27, *p* = 0.009). In multivariate analysis, the HER2/CEP17 ratio was an independent predictive factor for PFS (HR 0.66, *p* = 0.004) and potentially for OS (HR 0.64, *p* = 0.054), along with HER2 copy number (PFS HR 0.85, *p* = 0.004; OS HR 0.84, *p* = 0.049). Furthermore, the correlation between HER2 amplification level by ISH with PFS and OS was consistent across the HER2 IHC 1+/2+ and 3+ categories.

**Conclusions:**

This is the first study to report that a higher level of HER2 amplification by ISH is associated with improved PFS and OS in advanced HER2-positive breast cancer treated with dual HER2-blockade. Notably, HER2 amplification level had a predictive role regardless of IHC results. Even in patients with HER2 protein expression of 3+, treatment outcome to HER2-directed therapy was dependent on the level of HER2 gene amplification.

**Supplementary Information:**

The online version contains supplementary material available at 10.1186/s13058-023-01746-w.

## Introduction

Human epidermal growth factor receptor 2 (HER2) overexpression or amplification is found in 18–20% of invasive breast cancer and is associated with aggressive nature [1, 2]. The development of HER2 targeting agents over the last few decades has dramatically prolonged the survival outcome of patients with HER2-positive breast cancer [3, 4]. In the metastatic setting, dual HER2 blockade with trastuzumab and pertuzumab combined with docetaxel led to a greater survival benefit compared to single HER2 blockade with trastuzumab and docetaxel [5]. In the CLEOPATRA trial, pertuzumab, trastuzumab, and docetaxel showed a median overall survival (OS) of 56.5 months and have become the standard first-line treatment for HER2-positive metastatic breast cancer [6, 7].

HER2 positivity is defined as HER2 immunohistochemistry (IHC) 3+ or the presence of HER2 amplification by in-situ hybridization (ISH) [8, 9]. Although HER2 positivity is a strong predictive biomarker [10], some patients show primary resistance to HER2 targeting agents [11, 12]. More importantly, the duration of response to HER2-targeting agents varies from patient to patient [13]. Evidence shows that the level of HER2 amplification by ISH could be a useful predictive and prognostic factor for HER2-positive disease [14–16]. In the neoadjuvant setting, a higher HER2/CEP17 ratio was associated with a higher pathologic complete response (pCR) rate in patients treated with either a single or a dual HER2 blockade regimen [17–20]. In addition, higher HER2/CEP17 ratio was associated with higher objective response rate (ORR) and progression-free survival (PFS) in metastatic HER2-positive breast cancer treated with trastuzumab based regimen [16, 21]. However, there is no existing literature on the association between HER2 amplification level and treatment outcome in HER2-positive metastatic breast cancer treated with dual HER2 blockade, especially in those with HER2 IHC 3+. Although biomarker analysis in CLEOPATRA trial reported significantly improved PFS with high HER2 protein (IHC) and HER2 mRNA levels, the level of HER2 amplification was not evaluated, and HER2 mRNA levels are not readily available in clinical practice [22].

The purpose of this study was to evaluate the association between HER2 amplification level by ISH and treatment outcomes in patients with advanced HER2-positive breast cancer treated with first-line pertuzumab, trastuzumab, and docetaxel.

## Methods

### Study design and population

This retrospective cohort study included patients with locally advanced or metastatic HER2-positive breast cancer who had started palliative first-line treatment of pertuzumab, trastuzumab, and docetaxel at Seoul National University Hospital (SNUH, Seoul, Korea) between January 2014 and January 2021. Patients were eligible if they met the following criteria: age over 18 years; pathologically proven breast cancer; had archival tumor tissue taken prior to pertuzumab, trastuzumab, and docetaxel treatment; at least one measurable lesion; and at least two outpatient clinic visits after pertuzumab, trastuzumab, and docetaxel treatment. Eligible patients were identified from the electronic database and their medical charts were reviewed using the electronic medical record system of SNUH. Additional HER2 ISH staining of the archival tissue was performed in patients without previous HER2 ISH results. In patients with biopsies from multiple sites, the tissue selected for HER2 evaluation was based on the following criteria: for recurred cases, tissue from newly developed metastatic sites; for cases with discordant HER2 status, HER2-positive tissue; and when necessary, archival tissue of superior quality.

Each cycle of pertuzumab, trastuzumab, and docetaxel regimen consisted of tri-weekly administration of docetaxel 75 mg/m^2^ body surface area on day 1 of each cycle, trastuzumab 8 mg/kg administered on day 1 of cycle 1 followed by 6 mg/kg on day 1 of the remaining cycles, and pertuzumab 840 mg on day 1 of cycle 1 followed by 420 mg on day 1 of each subsequent cycle. Docetaxel was administered at the discretion of the treating physician with no limitation on the number of cycles.

The study protocol was reviewed and approved by the institutional review board of SNUH (H-2103-218-1210). This study was carried out in accordance with the recommendations of the Declaration of Helsinki for biomedical research involving human subjects. Informed patient consent was waived by the institutional review board of SNUH for posing minimal risk to the patients as this was a retrospective study.

### Analysis of tumor subtype and HER2 ISH

IHC staining for estrogen receptor (ER; clone 6F11; Novocastra Laboratories, Newcastle, UK), progesterone receptor (PR; clone 16; Novocastra Laboratories), HER2 (clone 4B5; Ventana Medical Systems, Tucson, AZ, USA), and Ki-67 (clone MIB-1; Dako/Agilent, Glostrup, Denmark) was performed on formalin-fixed, paraffin-embedded tissue. Hormone receptor (HR) status was considered positive if either ER or PR was positive (IHC ≥ 1%).

**Fig. 1:**
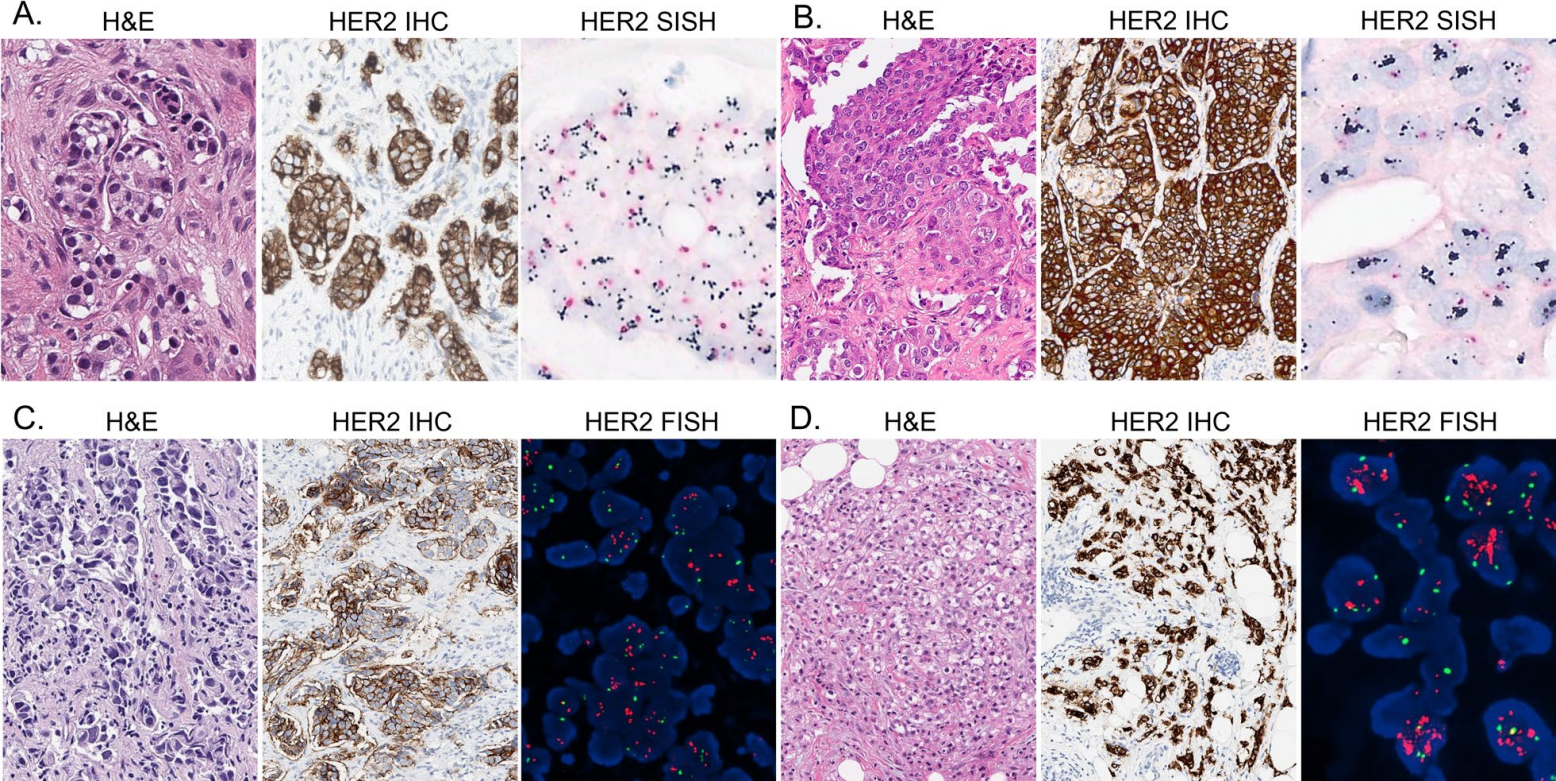
Representative results of HER2 immunohistochemistry, fluorescence and silver in situ hybridization. In each panel, representative figures of hematoxylin–eosin stain (H&E), HER2 immunohistochemistry (IHC) and *HER2* in situ hybridization (ISH) are presented from left to right. **A** Case with HER2 IHC of 3+, *HER2* copy number of 7.9, and *HER2/CEP17* ratio of 3.76. **B** Case with HER2 IHC of 3+, *HER2* copy number of 17.8, and *HER2/CEP17* ratio of 7.57. **C** Case with HER2 IHC of 2+, *HER2* copy number of 7.1, and *HER2/CEP17* ratio of 2.37. **D** Case with HER2 IHC of 3+, *HER2* copy number of 16.4, and *HER2/CEP17* ratio of 4.09. Note the higher *HER2* copy number and *HER2/CEP17* ratio in cases with HER2 IHC 3+ (panel **D**) compared to HER2 IHC 2+ (panel **C**)

The data including HER2 amplification level was collected from the pathology reports of the patients who had HER2 ISH results at the time of diagnosis. HER2 FISH was performed at the time of diagnosis using PathVysion HER-2 DNA Probe Kit (Abbott Molecular, Downers Grove, IL, USA). Additional SISH tests were performed using INFORM Her2 Dual ISH probe (Ventana Medical Systems) for HER2 3+ IHC patients who did not have reflex HER2 ISH results. When silver signals of HER2 were conglomerated as clusters, we interpreted small clusters as 6 copies, and large clusters were scored as 12 copies [23]. The representative results of HER2 IHC, FISH, and SISH are shown in Fig. 1.

All of the IHC and ISH testings were performed at the Laboratory of Immunohistochemistry and Molecular Pathology in SNUH, which is in charge of clinical biomarker assays of breast cancer including 4000+ cases of ER, PR, HER2 IHC and 500+ cases of HER2 ISH annually.

### Statistical analysis

The primary objective of this study was to investigate the predictive role of the HER2 amplification level on survival (PFS and OS) in advanced HER2 positive breast cancer patients treated with pertuzumab, trastuzumab, and docetaxel. The clinical database was last updated in April 2023. PFS was calculated from the first day of pertuzumab, trastuzumab, and docetaxel administration to disease progression or death, whichever occurred first. OS was calculated from the first day of pertuzumab, trastuzumab, and docetaxel administration to death from any cause. Treatment response was evaluated using RECIST criteria version 1.1.

Categorical variables were compared by chi-square test and continuous variables were compared using the independent-samples T test. The correlation between HER2 amplification level and survival (PFS and OS) was examined by Cox proportional hazard analysis. As HER2/CEP17 ratio and HER2 copy number did not show a normal distribution, statistical analysis was performed through a log transformation. PFS and OS was estimated using the Kaplan–Meier method and comparisons were made using the log-rank tests. Response rates were compared using the chi-square test. Multivariate analysis was performed with Cox proportional hazard regression model with a backward-stepwise method. To adjust for baseline characteristics, Cox proportional hazard analysis of PFS and OS included the following covariates: age, HR status, metastatic site, HER2 IHC, HER2/CEP17 ratio or HER2 copy number, and Ki-67 IHC. Two-sided *p* values of less than 0.05 were considered statistically significant. Statistical analysis was performed with R version 4.0.4 (www.r-project.org) using *survival* and *ggkm* packages and SPSS Statistics for Windows version 25.0 (IBM, Armonk, NY, USA).

## Results

### Patient characteristics

In total, 152 patients were included in this retrospective study. All patients were female with a median age of 53 years. The baseline characteristics are summarized in Table 1. Seventy patients (46.1%) had de novo metastatic disease and 82 (53.9%) had recurred disease. Visceral metastasis was found in 105 (69.1%) of the patients, while locally advanced tumor (unresectable) and bone-only metastases were found in 21 (13.8%) and 26 (17.1%), respectively. Liver, lung, and brain metastases were found in 45 (29.6%), 55 (36.2%), and 9 (5.9%) of the patients, respectively. Seventy-one (46.7%) patients had HR-positive HER2-positive disease and 81 (53.3%) patients had HR-negative HER2-positive disease. Among the 82 recurred cases, 74 (93.7%) patients received prior adjuvant or neoadjuvant chemotherapy. Prior treatment in 82 recurred patients includes anthracycline in 57 (69.5%), taxane in 49 (59.8%), and anti-HER2 therapy in 53 (64.6%). Adjuvant hormonal therapy was given to 44 (97.8%) of 45 initially HR-positive patients.

**Table 1:** General Characteristics

Characteristic	Total population (n = 152)	ISH evaluated population (n = 78)
Female sex—no. (%)	152 (100.0%)	78 (100.0%)
Age—year		
Median	53	58
Range	23–79	23–82
Disease status—no. (%)		
De novo metastatic disease	70 (46.1%)	31 (39.7%)
Recurrence	82 (53.9%)	47 (60.3%)
Metastatic site—no. (%)		
Locally advanced	21 (13.8%)	8 (10.2%)
Bone only	26 (17.1%)	13 (16.7%)
Visceral metastasis	105 (69.1%)	57 (73.1%)
Specific metastatic sites—no. (%)		
Liver	45 (29.6%)	24 (30.7%)
Lung	55 (36.2%)	31 (39.7%)
Brain	9 (5.9%)	4 (5.1%)
Hormone receptor status—no. (%)		
HR+ (ER+ or PR+)	71 (46.7%)	42 (53.8%)
HR− (ER− and PR−)	81 (53.3%)	36 (46.2%)
HER2 IHC—no. (%)		
1+	2 (1.3%)	2 (2.6%)
2+	21 (13.8%)	21 (26.9%)
3+	129 (84.9%)	55 (70.5%)
HER2/CEP17 ratio by ISH		
Median	3.23*	4.125
Range	1.6–8.89*	1.6–12.25
Prior adjuvant or neoadjuvant therapy among recurred disease—no. (%)	(n = 82)	(n = 47)
Chemotherapy	74 (93.7%)	45 (95.7%)
Anthracycline	57 (69.5%)	33 (70.2%)
Taxane	49 (59.8%)	32 (68.1%)
Anti-HER2	53 (64.6%)	31 (66.0%)
Hormonal therapy	44 (97.8%)	28 (100.0%)

*HR* hormone receptor, *ER* estrogen receptor, *PR* progesterone receptor, *HER2* human epidermal growth factor receptor 2, *IHC* immunohistochemistry, *CEP17* centromeric probe for chromosome 17, *ISH* in situ hybridization

* Only with those who had archival FISH results

**Fig. 2:**
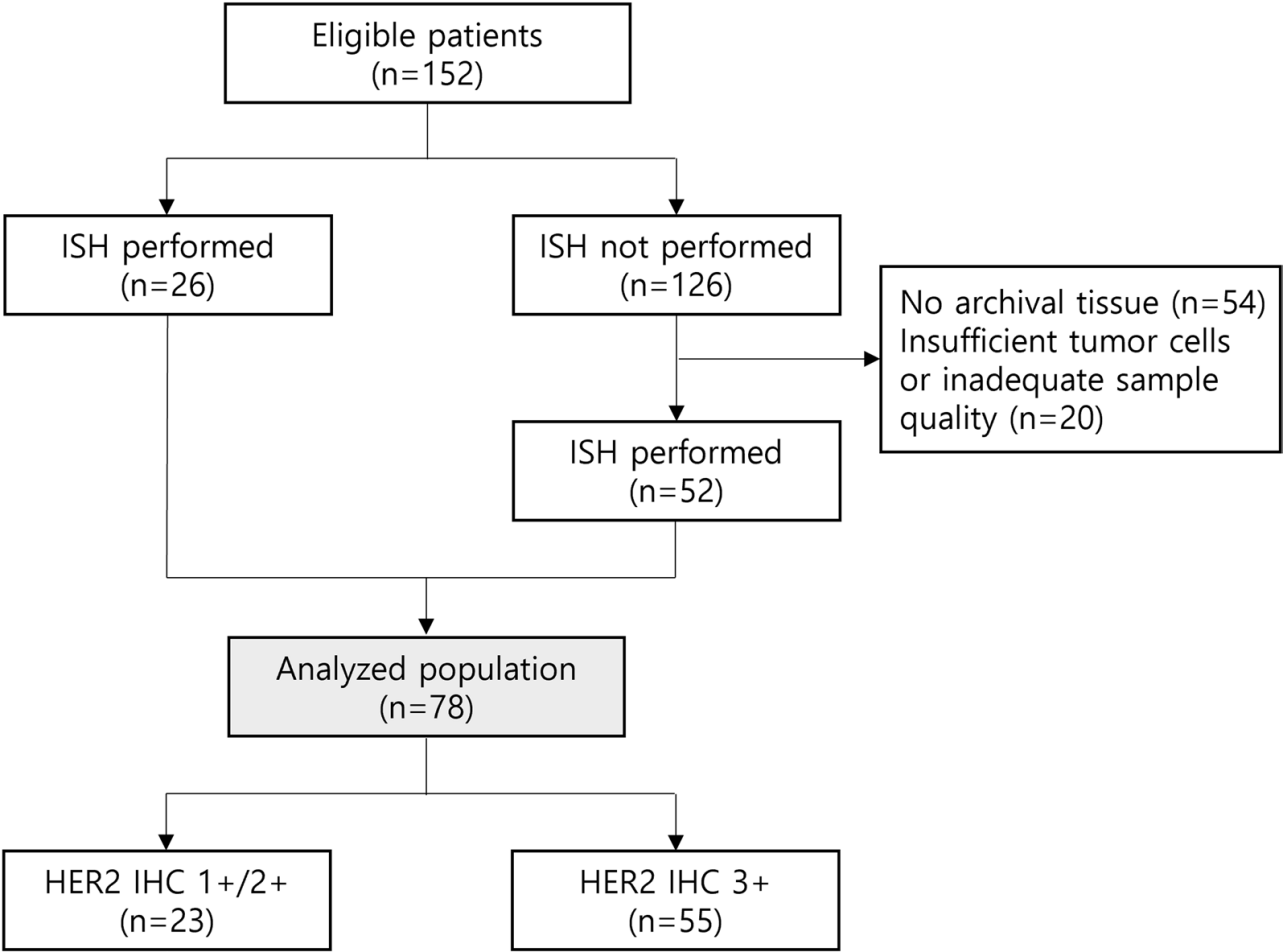
Study flow chart. HER2 ISH results at the time of diagnosis were available in 26 patients (2 patients with HER2 IHC 1+, 21 patients with 2+, and 3 patients with 3+ disease) with a median HER2/CEP17 ratio of 3.23 (ranged 1.6–8.89)

Tumor biopsy was taken before the initiation of pertuzumab, trastuzumab, and docetaxel in 143 (94.1%) patients out of 152. Out of 82 recurred patients, 73 patients (89.0%) had additional biopsy at the time of recurrence. For the remaining 9 patients (5.9%), biopsy was not clinically feasible, so initially obtained tissue from primary breast carcinoma was used. In the total population (n = 152), HER2 status was evaluated in the primary breast in 75 patients (49.3%), metastatic site in 58 (38.2%), and both in 19 (12.5%). 129 patients (84.9%) showed HER2 IHC of 3+, 21 (13.8%) had 2+, and 2 (1.3%) had 1+. HER2 ISH results at the time of diagnosis were available in 26 patients. Among the 126 patients without previous ISH results, all with HER2 IHC 3+, additional ISH testing was performed in 52 patients. Eventually, the level of HER2 amplification was analyzed in 78 patients with a median ratio of 4.13 (ranged 1.6–12.25). The general characteristics of these 78 patients are presented in Table 1 and Additional file 1: Table S1. Among them, 23 patients had HER2 IHC 1+ or 2+ and 55 patients had HER2 IHC 3+ (Fig. 2). All patients with IHC 3+ had HER2/CEP17 ratio greater than 2.0. Only one patient with IHC 2+ had an ISH ratio below 2.0 and was diagnosed as HER2 positive since her HER2 copy number was 7.75.

The treatment outcome of pertuzumab, trastuzumab, and docetaxel in 152 patients was as follows: ORR 78.3%, median PFS 21.9 months, and median OS 44.8 months. Docetaxel was administered for a median of 9 cycles, with a range of 2–40. This study cohort included 86 long-term responders whose PFS was longer than the median PFS from CLEOPATRA trial (18.7 months). The median PFS of these long-term responders was 41.4 months, ranging from 19.3 to 109.5 months. They received median 10 cycles of docetaxel (ranged 3–40) along with the maintenance of trastuzumab plus pertuzumab. Between long-term responders and non-long-term responders, there was no significant difference in age, metastatic site, hormone receptor status, or HER2 IHC. However, the HER2/CEP17 ratio of long-term responders (median 4.68, ranged 2.26–12.25) was significantly higher that of non-long-term responders (median 3.71, ranged 1.6–11.05) with a *p* value of 0.001.

### Predictive implication of HER2/CEP17 ratio

After a median follow-up duration of 50.0 months, 85 patients had disease progression and 27 had death events. Among the 78 patients with HER2 ISH results, 47 patients had disease progression and 19 patients had death events.

**Fig. 3:**
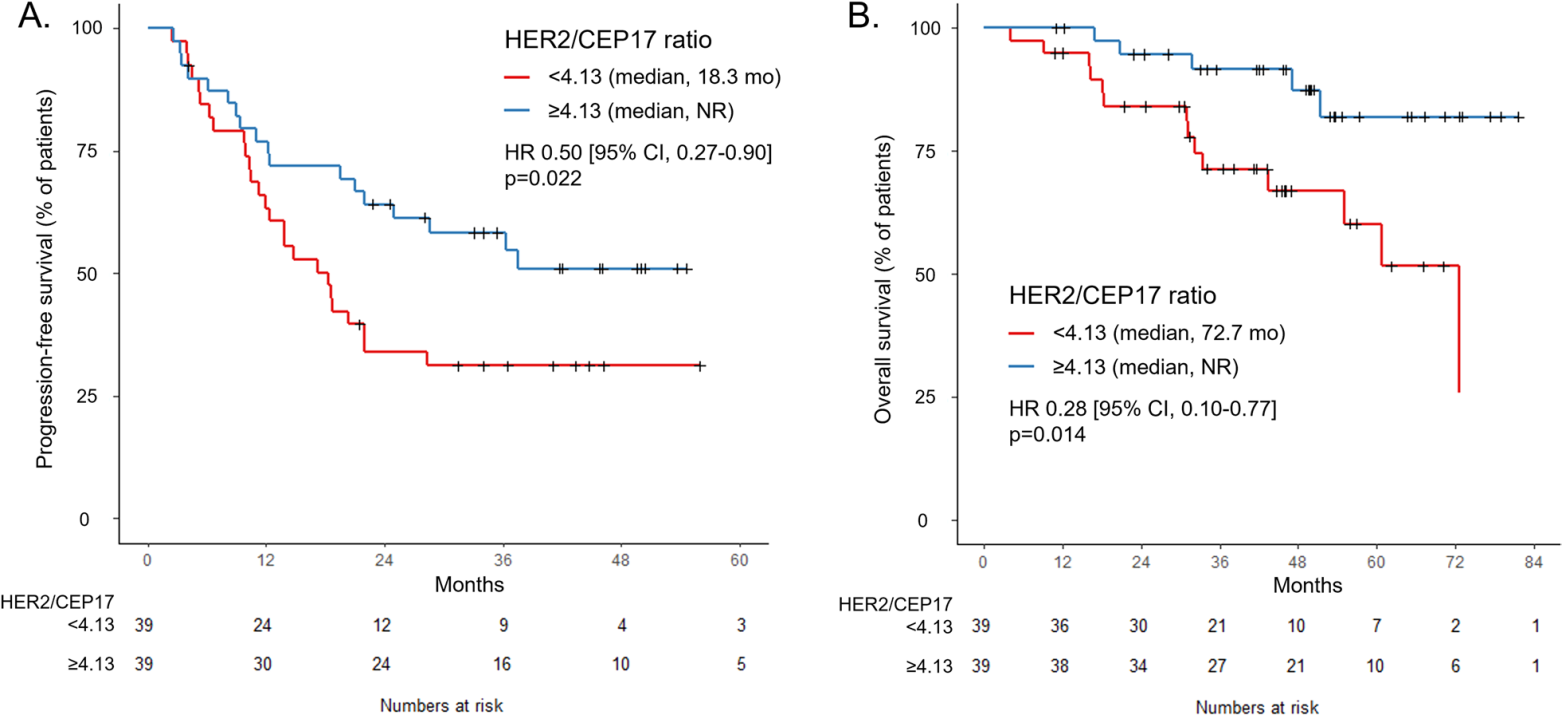
Treatment outcome according to HER2/CEP17 ratio. **A** Progression-free survival according to HER2/CEP17 ratio (n = 78). **B** Overall survival according to HER2/CEP17 ratio (n = 78)

The HER2/CEP17 ratio was significantly associated with PFS. Cox proportional hazard analysis revealed that the HER2/CEP17 ratio (in log transformation) was correlated with PFS (hazard ratio [HR] 0.21, 95% confidence interval [CI] 0.10–0.46, *p* < 0.001). When dichotomized by the median ISH ratio of 4.13, patients with a higher HER2/CEP17 ratio had longer PFS (median not reached versus 18.3 months, HR 0.50, 95% CI 0.27–0.90, *p* = 0.022) compared to those with lower HER2/CEP17 ratio (Fig. 3A). Objective response rate was numerically higher in patients with higher HER2/CEP17 ratio (ORR 76.9% vs. 69.2%) with an odds ratio of 1.47 (95% CI 0.48–4.65,* p* = 0.610).

**Fig. 4:**
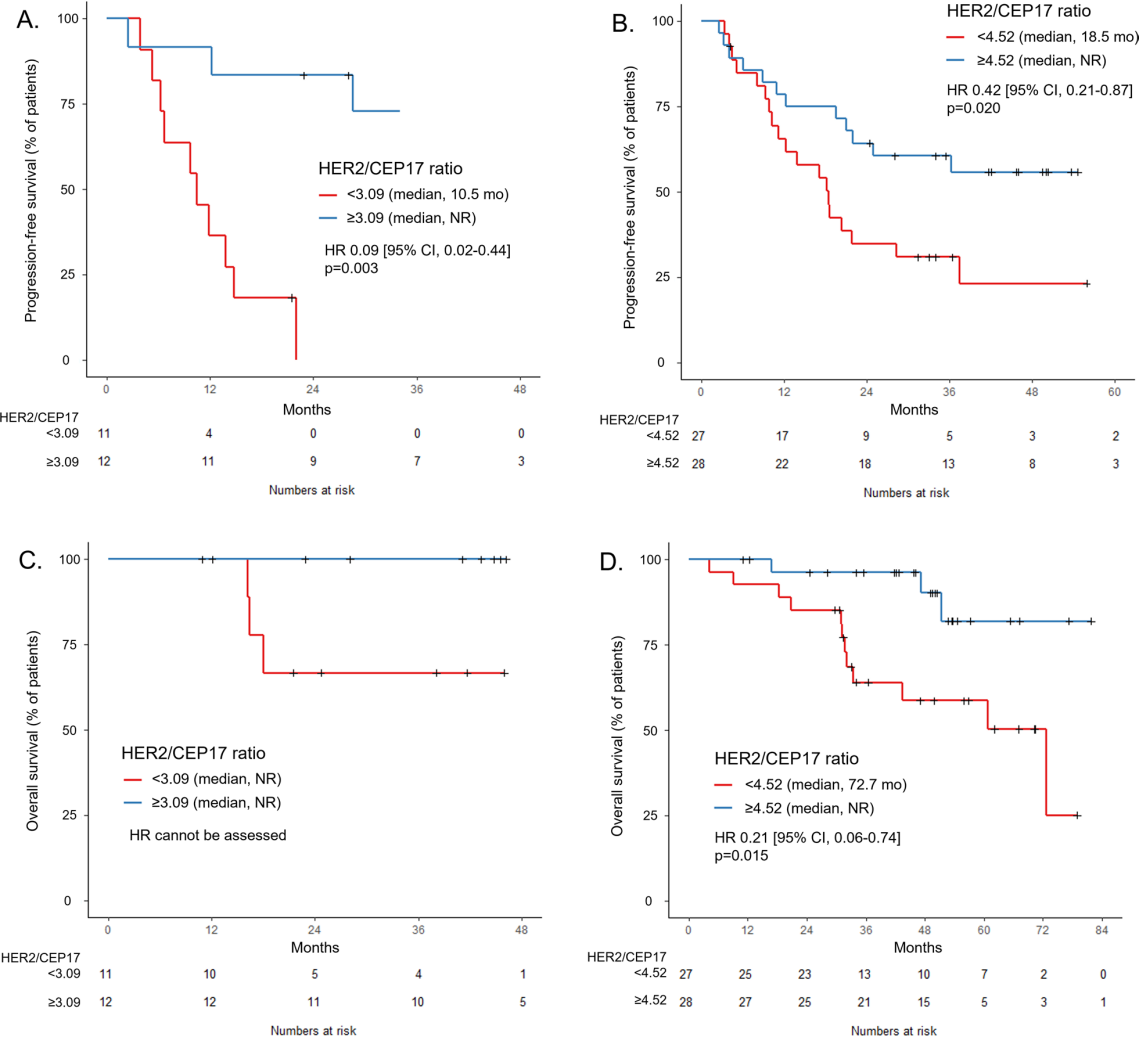
Treatment outcome based on HER2/CEP17 ratio and HER2 IHC. **A** Progression-free survival according to HER2/CEP17 ratio in HER2 IHC 1+/2+ (n = 23) and **B** HER2 IHC 3+ (n = 55). **C** Overall survival according to HER2/CEP17 ratio in HER2 IHC 1+/2+ (n = 23) and **D** HER2 IHC 3+ (n = 55)

The HER2/CEP17 ratio was associated with PFS in both HER2 IHC 1+/2+ (HR 0.08, 95% CI 0.01–0.55, *p* = 0.010) and HER2 IHC 3+ patients (HR 0.19, 95% CI 0.07–0.53, *p* = 0.001). When dichotomized by the median HER2/CEP17 ratio (3.09 for IHC 1+/2+, 4.52 for IHC 3+), patients with higher HER2/CEP17 ratio had longer PFS in both HER2 IHC 1+/2+ patients (median not reached versus 10.5 months, HR 0.09, 95% CI 0.02–0.44, *p* = 0.003) and HER2 IHC 3+ patients (median not reached versus 18.5 months, HR 0.42, 95% CI 0.21–0.87, *p* = 0.020) (Fig. 4A, B).

The factors associated with PFS were assessed using the Cox proportional hazard regression as shown in Table 2. In the univariate analyses, HER2/CEP17 ratio (HR 0.71, 95% CI 0.58–0.86, *p* = 0.001), and Ki-67 IHC ≥ 50% (HR 4.04, 95% CI 1.69–9.68, *p* = 0.002) were found to be statistically significant. Hormone receptor positivity (HR 0.88, 95% CI 0.57–1.35, *p* = 0.548), bone-only metastasis (HR 0.58, 95% CI 0.31–1.10, *p* = 0.098) and HER2 IHC 3+ (HR 0.84, 95% CI 0.47–1.50, *p* = 0.564) showed a tendency toward longer PFS, whereas age ≥ 50 (HR 1.35, 95% CI 0.86–2.11, *p* = 0.197) tended to have shorter PFS, although they failed to show statistical significance. In the multivariate analysis, only HER2/CEP17 ratio was associated with longer PFS (HR 0.66, 95% CI 0.49–0.88, *p* = 0.004).

**Table 2:** Proportional hazard regression of Progression-free survival

Variables	Univariate		Multivariate	
	HR [95% CI]	*p* value	HR [95% CI]	*p* value
Age ≥ 50 years	1.35 [0.86–2.11]	0.197		
HR+ (ER+ or PR+)	0.88 [0.57–1.35]	0.548		
Bone metastasis only	0.58 [0.31–1.10]	0.098		
HER2 IHC 3+ versus 1+/2+	0.84 [0.47–1.50]	0.564		
HER2/CEP17 ratio	0.71 [0.58–0.86]	0.001*	0.66 [0.49–0.88]	0.004*
Ki-67 IHC ≥ 50%	4.04 [1.69–9.68]	0.002*	1.49 [0.42–5.28]	0.533

*HR* hormone receptor, *ER* estrogen receptor, *PR* progesterone receptor, *HER2* human epidermal growth factor receptor 2, *IHC* immunohistochemistry, *CEP17* centromeric probe for chromosome 17, *HR* hazard ratio, *CI* confidence interval

* *P*-value < 0.05

The HER2/CEP17 ratio was also significantly associated with OS. Cox proportional hazard analysis revealed that the HER2/CEP17 ratio (in log transformation) showed a significant correlation with OS (HR 0.22, 95% CI 0.07–0.72, *p* = 0.012). When dichotomized by the median ISH ratio of 4.13, patients with a higher HER2/CEP17 ratio had longer OS (median not reached vs. 72.7 months, HR 0.28, 95% CI 0.10–0.77, *p* = 0.014) compared to those with lower HER2/CEP17 ratio (Fig. 3B). The HER2/CEP17 ratio showed a tendency of association with OS in both HER2 IHC 1+/2+ (HR 0.05, 95% CI 0.002–1.20, *p* = 0.064) and HER2 IHC 3+ patients (HR 0.15, 95% CI 0.03–0.79, *p* = 0.026). When dichotomized by the median HER2/CEP17 ratio (3.09 for IHC 1+/2+, 4.52 for IHC 3+), patients with higher HER2/CEP17 ratio had longer OS in both HER2 IHC 1+/2+ patients (median not reached, HR cannot be assessed) and HER2 IHC 3+ patients (median not reached vs. 72.7 months, HR 0.21, 95% CI 0.06–0.74, *p* = 0.015) (Fig. 4C, D). In addition, multivariate analysis by Cox proportional hazard regression indicated a trend in the HER2/CEP17 ratio (HR 0.64, 95% CI 0.41–1.01, *p* = 0.054) as a potential predictor of OS (Additional file 1: Table S2).

### Predictive implication of HER2 copy number

HER2 gene copy number showed similar predictive effects in treatment outcome. Cox proportional hazard analysis revealed that the HER2 copy number (in log transformation) was correlated with PFS (HR 0.24, 95% CI 0.12–0.46, *p* < 0.001) and OS (HR 0.27, 95% CI 0.10–0.72, *p* = 0.009). When dichotomized by its median, higher HER2 copy number was associated with longer PFS (HR 0.35, *p* < 0.001) and OS (HR 0.35, *p* = 0.035) (Additional file 2: Fig. S1). In addition, higher HER2 copy number was associated with longer PFS in both HER2 IHC 1+/2+ (HR 0.23, *p* = 0.020) and IHC 3+ (HR 0.31, *p* = 0.002) (Additional file 3: Fig. S2A, B). OS in HER2 IHC 1+/2+ group could not be analyzed due to insufficient number of death events, but OS in HER2 IHC 3+ group showed similar tendencies (HR 0.43, *p* = 0.127) (Additional file 3: Fig. S2C, D). In multivariate analysis, higher HER2 copy number was an independent predictive marker for PFS (HR 0.85, *p* = 0.004) (Additional file 1: Table S3) and OS (HR 0.84, *p* = 0.049) (Additional file 1: Table S4), when analyzed separately with HER2/CEP17 ratio as the two variables are correlated with each other.

## Discussion

To our knowledge, this is the first study to show that higher level of HER2 amplification by ISH is associated with favorable treatment outcomes in patients with advanced HER2-positive breast cancer treated with first-line pertuzumab, trastuzumab, and docetaxel. Higher HER2 amplification level was associated with a longer PFS and OS in both HER2 IHC 1+/2+ and IHC 3+ patients.

HER2 positivity, which is defined by HER2 IHC or ISH, is a strong predictive biomarker for HER2-directed therapy [10]. However, the duration of response to HER2-directed therapy varies from patient to patient as some patients show primary resistance to anti-HER2 therapy [13]. The magnitude of HER2 amplification has been proposed as a potential predictive marker for HER2-directed therapy [14, 24]. In the neoadjuvant setting, HER2/CEP17 ratio ≥ 4.5 (OR 2.11, *p* = 0.005) [20] or > 6 (pCR rate 69.0% vs. 30.4%, *p* = 0.001) [19] was associated with a higher pCR for trastuzumab-based single blockade. This finding was also shown in early HER2 positive breast cancer treated with dual HER2 blockade (pCR vs. non-pCR, median HER2/CEP17 ratio 7.08 vs. 4.70, *p* = 0.030) [18]. A linear relationship between the HER2/CEP17 ratio and pCR was reported (pCR rate according to HER2/CEP17 ratio in quartiles: 20%, 33%, 44%, and 56%) but was statistically insignificant [17]. In the palliative setting, HER2/CEP17 ratio ≥ 3.0 was associated with longer PFS (median 17.2 vs. 7.4 months, *p* = 0.002) for single HER2 blockade [16]. As for dual HER2 blockade, biomarker analysis from CLEOPATRA study revealed that HER2 protein (*p* = 0.05) and HER2 mRNA (*p* = 0.008) were significant predictive variables for PFS [22]. However, to our knowledge, no study has evaluated the predictive role of HER2 amplification level in advanced HER2-positive breast cancer treated with dual HER2 blockade.

In this study, patients with high HER2/CEP17 ratio had longer PFS (median not reached vs. 18.3 months, HR 0.50, *p* = 0.022), longer OS (median not reached vs. 72.7 months, HR 0.28, *p* = 0.014), and numerically higher ORR (76.9% vs. 69.2%, *p* = 0.610) compared to those with low HER2/CEP17 ratio. Higher HER2/CEP17 ratio was associated with longer PFS and OS in both HER2 IHC 1+/2+ patients and HER2 IHC 3+ patients. In the multivariate analysis, the HER2/CEP17 ratio was an independent predictive marker of PFS (HR 0.66, *p* = 0.004) and a potential predictor of OS (HR 0.64, *p* = 0.054), while age, HR status, metastatic site, and Ki-67 IHC failed to show predictive implications. Higher HER2 copy number was also a predictive biomarker for better PFS and OS.

The strength of our study is that we performed additional ISH in all HER2 IHC 3+ patients with available samples and thus were able to evaluate the predictive role of the HER2 amplification level in patients of all HER2 IHC status. In the clinical practice, HER2 ISH is usually performed as a reflex testing for those with HER2 IHC 2+ and is seldom assessed in patients with HER2 IHC 3+, because these patients are regarded as HER2-positive irrespective of ISH results [9]. Therefore, retrospective studies on HER2 amplification level tend to involve more patients with HER2 IHC 2+ which do not represent the entire spectrum of HER2-positive disease [16]. In the present study, we performed additional ISH in 52 patients with HER2 IHC 3+, providing novel information regarding the HER2 amplification level within this population.

We also evaluated the clinical implications of other clinicopathologic factors. HR positivity and bone-only metastasis showed a tendency toward longer PFS, whereas age ≥ 50 and Ki-67 IHC ≥ 50% tended to have shorter PFS, yet statistically insignificant. However, multivariate analyses showed that only HER2/CEP17 ratio and HER2 copy number was an independent predictive factor for PFS.

This study cohort showed comparable treatment outcomes to CLEOPATRA trial. With pertuzumab, trastuzumab, and docetaxel treatment, patients showed an ORR of 78.3%, median PFS of 21.9 months, and median OS of 44.8 months. Meanwhile, it included 86 long-term responders whose PFS was longer than the median PFS from CLEOPATRA trial (18.7 months). The HER2/CEP17 ratio of long-term responders were significantly higher than non-long-term responders (median 4.68 vs. 3.71, *p* = 0.001).

This study focused on the quantitative evaluation of HER2 by ISH testing. HER2 proteins are the main targets for anti-HER2 therapy, thus HER2 protein expression assessed by IHC has been widely used to predict the treatment response. One of the caveats of IHC, however, is that its interpretation still remains at the level of “semi”-quantification, that is, we cannot discriminate the relative abundance of the HER2 protein within the HER2 IHC 3+ cases or HER2 IHC 2+ categories. In this perspective, we hypothesized that HER2/CEP17 ratios and HER2 copy numbers by ISH may give higher resolution and further information within each of the IHC categories. As a result, we found that quantitatively assessed HER2/CEP17 ratios and HER2 gene copy numbers have additional values for predictive stratification of the patients receiving anti-HER2 therapies. Though gene amplification status may not always represent the overexpression at the protein level, our data strongly suggest the additional benefit of continuous, quantitative data provided by HER2 ISH for prediction, which was not readily achievable by IHC alone.

Antibody–drug conjugates, such as trastuzumab deruxtecan (T-DXd), have recently advanced the treatment of HER2+ metastatic breast cancer. T-DXd is being compared to dual HER2 blockade in the first-line setting with DESTINY-Breast09 (NCT04784715). Looking at the significant proportion of long-term responders to dual HER2 blockade in this study, it is crucial to identify which patients would benefit sufficiently from standard dual HER2 blockade and which should be candidates for upfront T-DXd. HER2/CEP17 ratio as well as HER2 copy number and HER2 protein expression should be examined as one of the potential predictive biomarkers for such. In addition, DESTINY-Breast04 has proved the efficacy of T-DXd in previously treated HER2-low breast cancer [25]. Along with other attempts to diagnose HER2-low disease by quantitatively measuring the level of HER2 protein expression, HER2/CEP17 ratio and HER2 copy number may also be evaluated to explore their potential diagnostic or predictive role in HER2-low tumor.

A limitation of this study is the incomplete availability of pre-treatment biopsies for all patients receiving palliative pertuzumab, trastuzumab, and docetaxel. However, it is noteworthy that HER2 status was assessed at that stage for 91.5% of the patients.

In conclusion, a higher level of HER2 amplification by ISH was associated with a longer PFS and OS in HER2-positive advanced breast cancer patients treated with first-line pertuzumab, trastuzumab, and docetaxel. Even in patients with HER2 protein expression of 3+, the treatment outcome to HER2-directed therapy was dependent on the level of HER2 gene amplification. It would be helpful to perform ISH not only in patients whose HER2 IHC result is ambiguous, but also in patients with HER2 IHC 3+ to improve the prediction of treatment outcomes.

### Supplementary Information

**Additional file 1: Table S1.** General characteristics according to HER2/CEP17 ratio. **Table S2.** Proportional hazard regression of overall survival regarding HER2/CEP17 ratio. **Table S3.** Proportional hazard regression of progression-free survival regarding HER2 copy number. **Table S4.** Proportional hazard regression of overall survival regarding HER2 copy number.

**Additional file 2.** **Figure S1**. Treatment outcome according to HER2 copy number. (A) Progression-free survival and (B) Overall survival, dichotomized by the median HER2 copy number of 10.0.

**Additional file 3.** **Figure S2.** Treatment outcome based on HER2 copy number and HER2 IHC. (A) Progression-free survival in HER2 IHC 1+/2+ and (B) HER2 IHC 3+. (C) Overall survival in HER2 IHC 1+/2+ and (D) HER2 IHC 3+ (dichotomized by the respective median HER2 copy number)

## Data Availability

The datasets used and/or analyzed during the current study are available from the corresponding author on reasonable request.
