# Supplementary material for: HER2 amplification level by in situ hybridization predicts survival outcome in advanced HER2-positive breast cancer treated with pertuzumab, trastuzumab, and docetaxel regardless of HER2 IHC results
Source: Breast Cancer Res. 2023 Dec 14;25:154. doi: 10.1186/s13058-023-01746-w (PMC10722732; doi:10.1186/s13058-023-01746-w)
Supplement: Supplementary file 1 — Additional file 1: Table S1. General characteristics according to HER2/CEP17 ratio. Table S2. Proportional hazard regression of overall survival regarding HER2/CEP17 ratio. Table S3. Proportional hazard regression of progression-free survival regarding HER2 copy number. Table S4. Proportional hazard regression of overall survival regarding HER2 copy number. [file 13058_2023_1746_MOESM1_ESM.docx]

**Table S1. General Characteristics according to HER2/CEP17 ratio.**

| **Characteristic** | **HER2/CEP17**  **<4.13**  **(n=39)** | **HER2/CEP17**  **≥4.13**  **(n=39)** | ***P* value** |
| --- | --- | --- | --- |
| **Age – yr** |  |  | 0.425 |
| Median | 55 | 52 |  |
| Range | 23 – 79 | 38 – 76 |  |
| **Disease status – no. (%)** |  |  | 0.644 |
| De novo metastatic disease | 17 (43.6%) | 14 (35.9%) |  |
| Recurrence | 22 (56.4%) | 25 (64.1%) |  |
| **Metastatic site – no. (%)** |  |  | 0.654 |
| Locally advanced | 4 (10.3%) | 4 (10.3%) |  |
| Bone only | 8 (20.5%) | 5 (12.8%) |  |
| Visceral metastasis | 27 (69.2%) | 30 (76.9%) |  |
| **Specific metastatic sites – no. (%)** |  |  |  |
| Liver | 12 (30.8%) | 12 (30.8%) | 1.000 |
| Lung | 12 (30.8%) | 19 (48.7%) | 0.165 |
| Brain | 3 (7.7%) | 1 (2.6%) | 0.608 |
| **Hormone receptor status – no. (%)** |  |  | 0.820 |
| HR+ (ER+ or PR+) | 22 (56.4%) | 20 (51.3%) |  |
| HR- (ER- and PR-) | 17 (43.6%) | 19 (48.7%) |  |
| **HER2 IHC – no. (%)** |  |  | 0.004 |
| 1+ | 1 (2.6%) | 1 (2.6%) |  |
| 2+ | 17 (43.6%) | 4 (10.3%) |  |
| 3+ | 21 (53.8%) | 34 (87.2%) |  |
| **HER2/CEP17 ratio by ISH** |  |  | <0.001 |
| Median | 4.13 | 5.51 |  |
| Range | 1.60 – 4.12 | 4.13 – 12.25 |  |

Abbreviations: HR, hormone receptor; ER, estrogen receptor; PR progesterone receptor; HER2, human epidermal growth factor receptor 2; IHC, immunohistochemistry; CEP17, centromeric probe for chromosome 17; ISH, in situ hybridization

**Table S2.** **Proportional hazard regression of Overall survival regarding HER2/CEP17 ratio.**

| **Variables** | **Univariate** | | **Multivariate** | |
| --- | --- | --- | --- | --- |
|  | **HR [95% CI]** | **P value** | **HR [95% CI]** | **P value** |
| Age ≥50yr | 1.76 [0.77-4.03] | 0.182 |  |  |
| HR+ (ER+ or PR+) | 0.86 [0.40-1.83] | 0.692 |  |  |
| Bone metastasis only | 0.70 [0.24-2.02] | 0.504 |  |  |
| HER2 IHC 3+ vs 1+/2+ | 0.90 [0.31-2.61] | 0.850 |  |  |
| HER2/CEP17 ratio | 0.71 [0.52-0.97] | 0.033* | 0.64 [0.41-1.01] | 0.054 |
| Ki-67 IHC ≥50% | 5.58 [1.59-19.59] | 0.007* | 3.16 [0.62-16.07] | 0.166 |

Abbreviations: HR, hormone receptor; ER, estrogen receptor; PR progesterone receptor; HER2, human epidermal growth factor receptor 2; IHC, immunohistochemistry; CEP17, centromeric probe for chromosome 17; HR, hazard ratio; CI, confidence interval

**Table S3. Proportional hazard regression of Progression-free survival regarding HER2 copy number.**

| **Variables** | **Univariate** | | **Multivariate** | |
| --- | --- | --- | --- | --- |
|  | **HR [95% CI]** | **P value** | **HR [95% CI]** | **P value** |
| Age ≥50yr | 1.35 [0.86-2.11] | 0.197 |  |  |
| HR+ (ER+ or PR+) | 0.88 [0.57-1.35] | 0.548 |  |  |
| Bone metastasis only | 0.58 [0.31-1.10] | 0.098 |  |  |
| HER2 IHC 3+ vs 1+/2+ | 0.84 [0.47-1.50] | 0.564 |  |  |
| HER2 copy number | 0.85 [0.78-0.92] | <0.001* | 0.85 [0.76-0.95] | 0.004* |
| Ki-67 IHC ≥50% | 4.04 [1.69-9.68] | 0.002* | 1.15 [0.31-4.32] | 0.839 |

Abbreviations: HR, hormone receptor; ER, estrogen receptor; PR progesterone receptor; HER2, human epidermal growth factor receptor 2; IHC, immunohistochemistry; CEP17, centromeric probe for chromosome 17; HR, hazard ratio; CI, confidence interval

**Table S4. Proportional hazard regression of Overall survival regarding HER2 copy number.**

| **Variables** | **Univariate** | | **Multivariate** | |
| --- | --- | --- | --- | --- |
|  | **HR [95% CI]** | **P value** | **HR [95% CI]** | **P value** |
| Age ≥50yr | 1.76 [0.77-4.03] | 0.182 |  |  |
| HR+ (ER+ or PR+) | 0.86 [0.40-1.83] | 0.692 |  |  |
| Bone metastasis only | 0.70 [0.24-2.02] | 0.504 |  |  |
| HER2 IHC 3+ vs 1+/2+ | 0.90 [0.31-2.61] | 0.850 |  |  |
| HER2 copy number | 0.86 [0.75-0.98] | 0.021* | 0.84 [0.71-1.00] | 0.049* |
| Ki-67 IHC ≥50% | 5.58 [1.59-19.59] | 0.007* | 2.32 [0.40-13.29] | 0.345 |

Abbreviations: HR, hormone receptor; ER, estrogen receptor; PR progesterone receptor; HER2, human epidermal growth factor receptor 2; IHC, immunohistochemistry; CEP17, centromeric probe for chromosome 17; HR, hazard ratio; CI, confidence interval
