# Supplementary figures and images for: HER2 amplification level by in situ hybridization predicts survival outcome in advanced HER2-positive breast cancer treated with pertuzumab, trastuzumab, and docetaxel regardless of HER2 IHC results
Source: Breast Cancer Res. 2023 Dec 14;25:154. doi: 10.1186/s13058-023-01746-w (PMC10722732; doi:10.1186/s13058-023-01746-w)

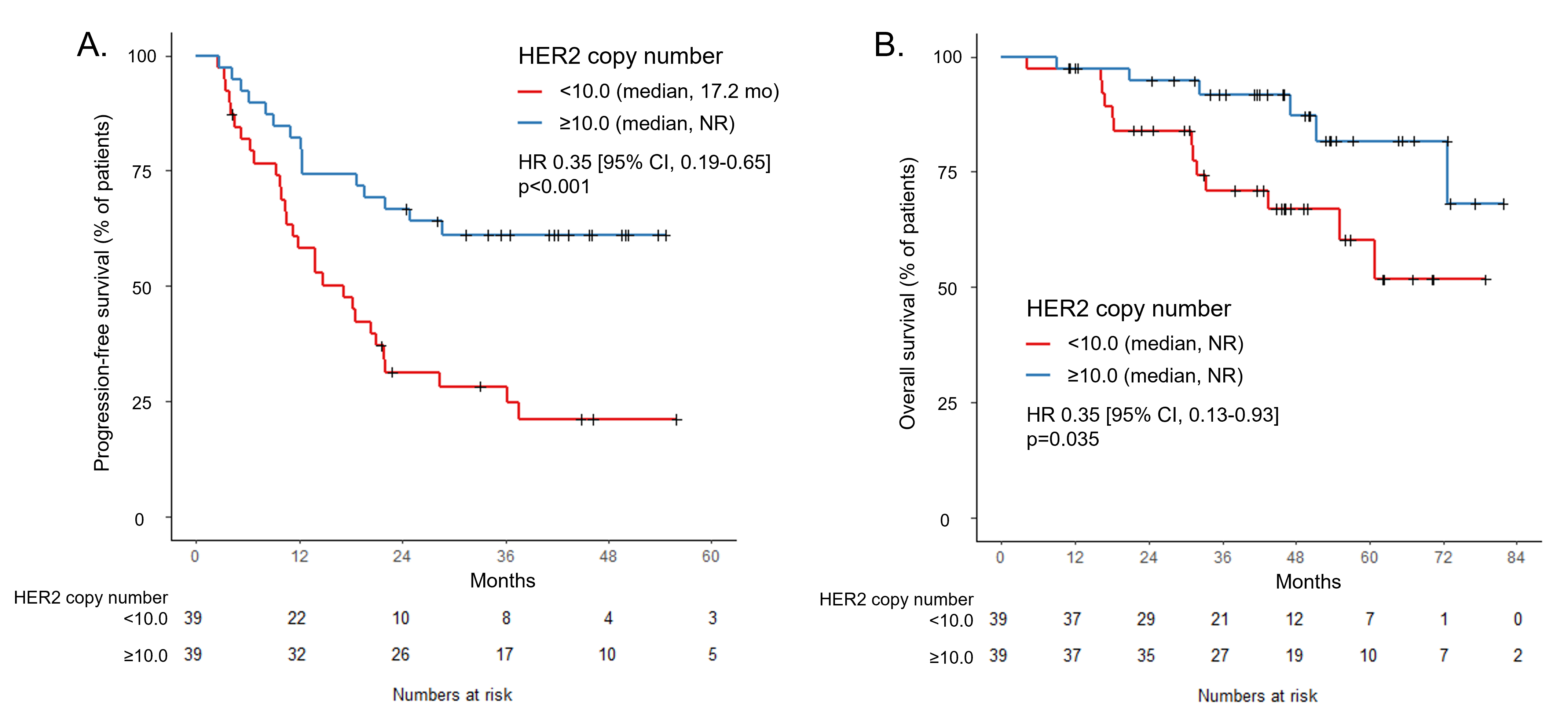

Supplement: Supplementary file 2 — Additional file 2. Figure S1. Treatment outcome according to HER2 copy number. (A) Progression-free survival and (B) Overall survival, dichotomized by the median HER2 copy number of 10.0. [file 13058_2023_1746_MOESM2_ESM.tif]

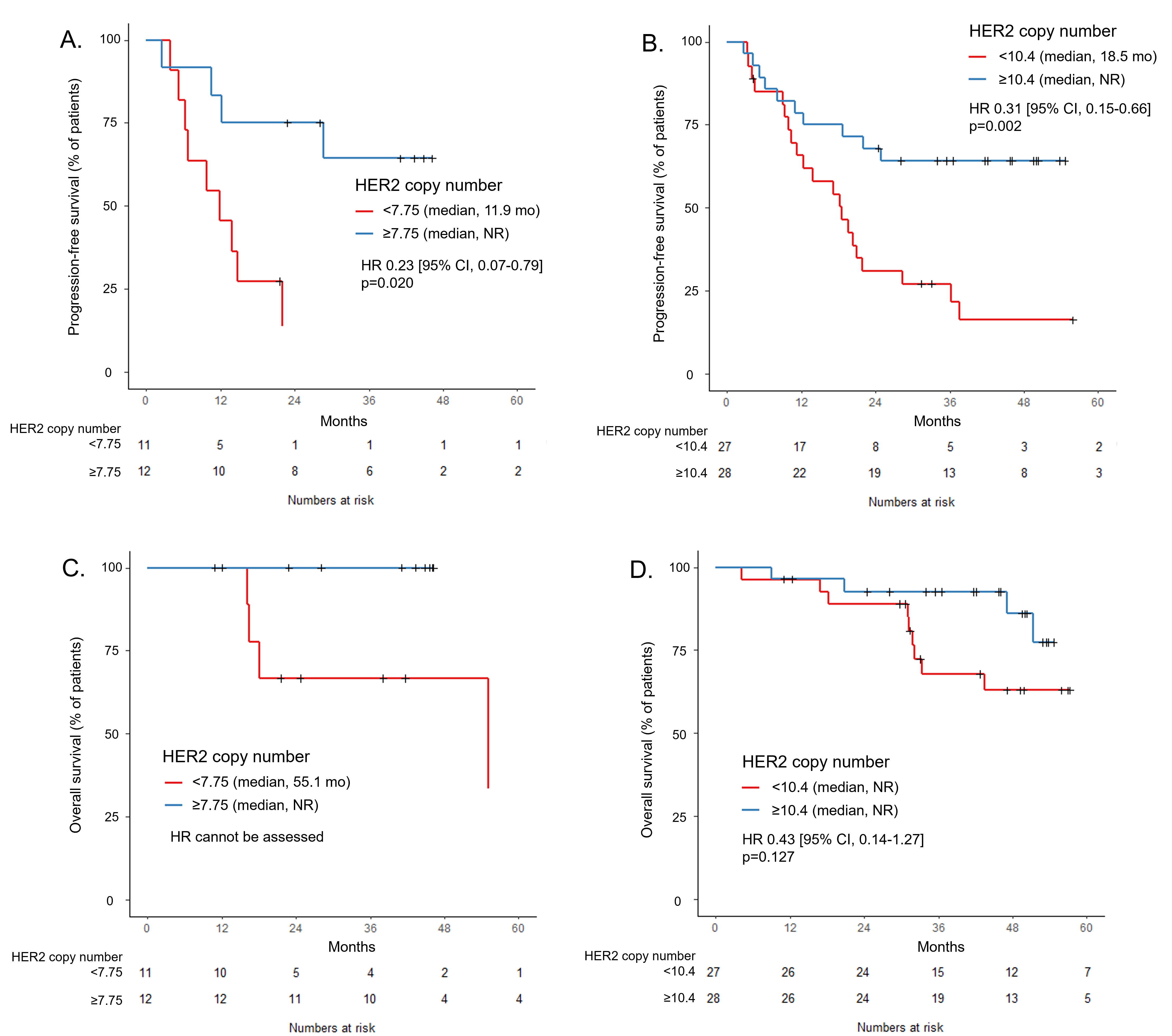

Supplement: Supplementary file 3 — Additional file 3. Figure S2. Treatment outcome based on HER2 copy number and HER2 IHC. (A) Progression-free survival in HER2 IHC 1+/2+ and (B) HER2 IHC 3+. (C) Overall survival in HER2 IHC 1+/2+ and (D) HER2 IHC 3+ (dichotomized by the respective median HER2 copy number) [file 13058_2023_1746_MOESM3_ESM.tif]
